# Development and application of a rapid *Mycobacterium tuberculosis* detection technique using polymerase spiral reaction

**DOI:** 10.1038/s41598-018-21376-z

**Published:** 2018-02-14

**Authors:** Wei Liu, Dayang Zou, Xiaoming He, Da Ao, Yuxin Su, Zhan Yang, Simo Huang, Qinghe Zhao, Yue Tang, Wen Ma, Yongfeng Lu, Jing Wang, Xinjing Wang, Liuyu Huang

**Affiliations:** 1 The Institute for Disease Prevention and Control of PLA, Beijing, 100071 China; 2 Clinical Laboratory, Institute of Tuberculosis, 309 hospital of PLA, Beijing, 100071 China; 3 Chinese academy of inspection and quarantine, Beijing, 100176 China; 4 Institute for Drug and Instrument Control of Health Dept GLD of PLA, Beijing, 100071 China; 5 The Department of Obstetrics and Gynecology, The Second Affiliated Hospital, Zhengzhou University, Zhengzhou, 450014 China

## Abstract

*Mycobacterium tuberculosis* is an age-old bacterium that is difficult to eliminate. A simple and rapid diagnostic method is of great importance to prevent the spread of *M*. *tuberculosis*. Here, we developed a low-cost rapid *M*. *tuberculosis* nucleic acid detection technique, named GenePop, which enabled the storage and transport of *M*. *tuberculosis* diagnostic reagent at ambient temperatures, without the need for professional operations or expensive instrumentation. Using a vitrification method, we vitrified heat-unstable components onto the cap of a reaction tube, and placed heat-stable components at the bottom of the reaction tube by sealing them with paraffin wax. The all-in-one detection tube, when used together with our other invention—a multi-functional sample treatment tube pre-filled with a nucleic acid-releasing agent—only required three simple steps to yield results. A comparative analysis with a commercial qPCR kit for *M*. *tuberculosis* indicated that our new technique had a concordance rate of 91.6%, showing no cross-reactivity with 11 other bacteria. The complete operation time was only 65 min. It is suitable for use in field settings or by personnel in grass-root units, and is applicable in household activities, hence can be used in developing countries.

## Introduction

Studies have shown that the prevalence of tuberculosis in humans can be dated back to 500,000 years^1^. This age-old disease causes death at a rate that ranks second among death rates caused by diseases from single infections^2^. Tuberculosis is a chronic infectious disease caused by *Mycobacterium tuberculosis*. In 2015, 10.4 million people were infected with *M*. *tuberculosis*, and 1.4 million deaths were recorded due to this disease (WHO, 2016). China is one of the 22 countries with high prevalence of tuberculosis, and approximately one third of the Chinese population—more than 400 million (active and latent TB) — are infected with *M*. *tuberculosis*. This disease has a prolonged treatment cycle, and the drugs used have severe toxic side effects; moreover, the emergence of drug resistant and multi-drug resistant bacteria makes it very difficult to cure patients with tuberculosis. Therefore, a rapid, specific, and sensitive *M*. *tuberculosis* detection method that would benefit diagnosis and treatment of this disease should be developed urgently, and it would be the prerequisite for preventing the spread of this disease when close contacts were diagnosed, quarantined, and treated in a timely manner.

Currently, microscopic observation and culture remain the predominant detection method for *M*. *tuberculosis* in several countries (WHO, 2008), but microscopy has a low sensitivity, needs multiple sampling from a patient, and requires repeated tests. In contrast to the microscopic detection method, detection based on sputum cultivation has 100-times higher sensitivity^3^ and high specificity; therefore, it has been the gold standard for the diagnosis of tuberculosis until date. However, from the perspective of clinical application, the growth of *M*. *tuberculosis* isolated from sputum in culture medium is slow with cultivation time up to 1–2 months in the traditional Löwenstein–Jensen medium^4^. Therefore, in the fight against tuberculosis worldwide, development of a simple, rapid, and efficient diagnostic method for this disease is a key issue^5^. PCR for *M*. *tuberculosis* based on gene therapy addresses the above issue to some extent^6,7^, showing a certain degree of increase in the detection sensitivity and specificity. However, the complete process of PCR takes around 3–5 h, and needs professional, well-trained personnel to perform the test in a specific experimental setting at a precisely controlled temperature. Moreover, some researchers have tried employing nested PCR or real-time PCR for the rapid detection of *M*. *tuberculosis*^8,9^, which showed a certain degree of increase in the detection specificity, but the processes required expensive instrumentations and thereby failed to fulfill the demand for a simple and rapid detection.

*M*. *tuberculosis* nucleic acid detection requires the following: a kit to extract nucleic acids from samples; a low temperature refrigerator to store heat-unstable components; a cold-chain transport to ensure the activity of heat-unstable components; precise pipettes for preparing a reaction system; well-trained personnel to perform the test operations; and precise and expensive instruments for the test. Instead of fulfilling the above-mentioned rigorous requirements, we aimed to make the diagnosis of tuberculosis easier to perform by the general public. For this, we invented a nucleic acid extraction device to overcome limitations of the regular nucleic acid kits, employed a vitrification method to overcome the ambient temperature maintenance problem for heat-unstable components, used an all-in-one package containing all the agents to solve the problems associated with sample introduction and PCR product contamination, and employed isothermal polymerase spiral reaction (PSR) method, invented by us, for nucleic acid testing, thereby circumventing the requirement of precise and expensive instruments.With all these innovations, we successfully established a low-cost, rapid, portable nucleic acid test platform, which does not require low temperature storage, professional operation skills, or precise and expensive instruments, making this method suitable for rapid nucleic acid testing in field settings or by staff of grass-root units and also for household use. We named this method GenePop.

## Methods and Results

### Rapid extraction of nucleic acids from *M*. *tuberculosis* from sputum samples

Nucleic acid extraction is the most basic and important step in molecular biology experiments. Currently, there are several types of nucleic acid extraction methods with various degrees of complexity. Conducting experiments on molecular diagnosis, nucleic acid sequencing, and functional genomics relies on successful nucleic acid extraction. Until now, there are two types of kits available for nucleic acid extraction commercially—one is to be used in automatic nucleic acid extractors, which are expensive and large in volume, need to be maintained by trained personnel, and primarily used by large hospitals and national laboratories; the other is more regular, but the process involves centrifugation, is tedious in operation, and depends highly on professional skills, and therefore, is mainly used in research institutions. We developed a multi-functional sample treatment device to fulfil the requirements for nucleic acid extraction. The device was easy to operate with low requirement for trained personnel—without the need for an instrument, a pipette, or a pre-prepared solution, and could be stored at ambient temperatures without cross-contamination. With high extraction purity, the complete process of DNA extraction from a sputum sample could be achieved within 15 min.

The multi-functional sample treatment tube, designed witha unique perspective, was filled with an optimized lysis solution that could quickly lyse *M*. *tuberculosis* cells to release nucleic acids. The lysis solution used in this step could also be used in subsequent nucleic acid amplification reactions, including regular PCR, quantitative fluorescence PCR, PSR, and LAMP isothermal amplification. The complete extraction process did not involve complicated experimental steps, such as centrifugation, sample introduction, and removal of proteins and other secondary metabolites, and did not include organic solvent extraction or anhydrous ethanol precipitation, thereby saving experimentation time and reducing cross-contamination among samples.

**Figure 1:**
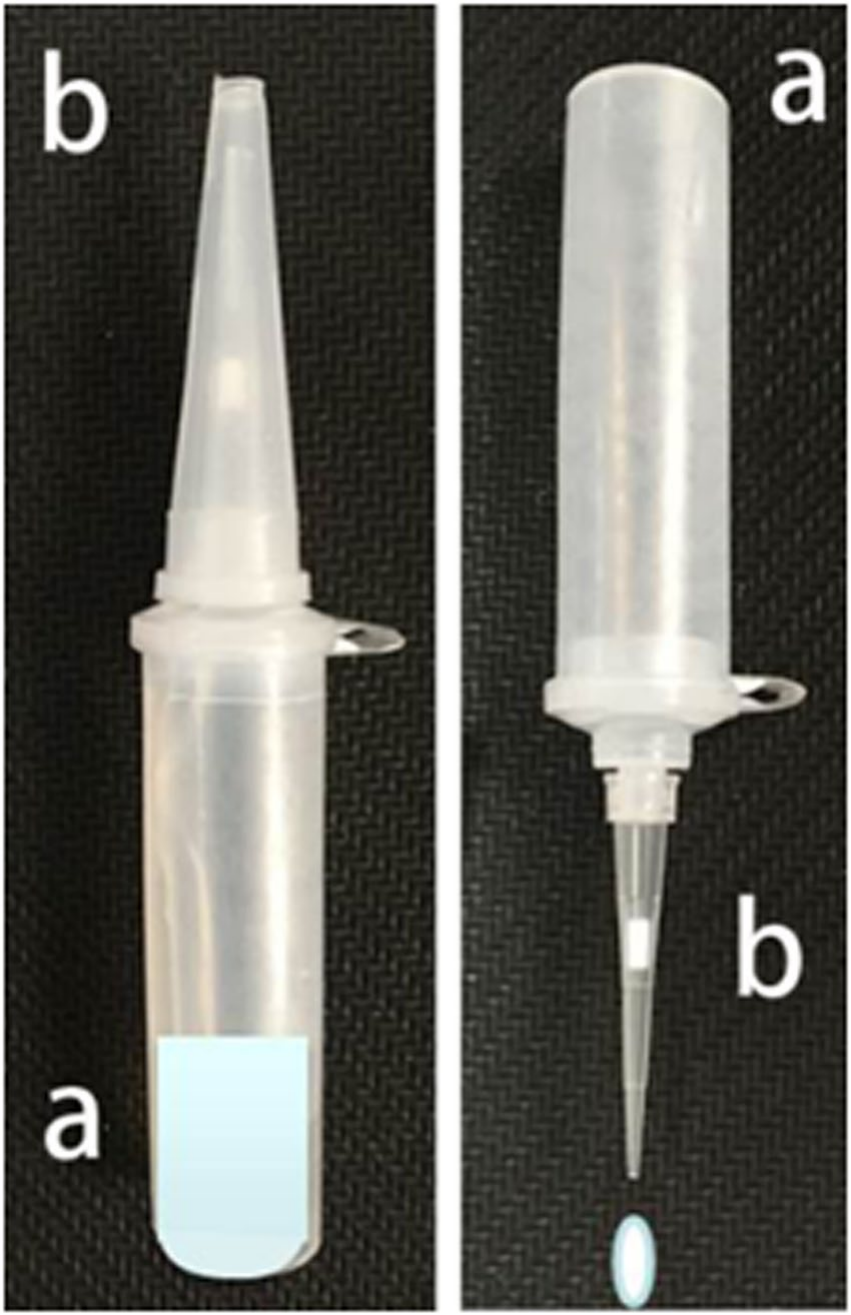
A multi-functional sample treatment device.

The tube had a special design as shown in Fig. 1. The tube contained two ends—‘a’ and ‘b’. The end ‘a’ was the heating end where a regular lysis solution was placed, while the end ‘b’ was a sample introduction end where a filtration membrane was fitted on the top that could filter out impurities in the sample or particulates in the lysis solution. Moreover, in the middle of the end ‘b’, there was a sample introduction section, which could be opened manually with force by the personnel for squeezing out sample droplets and closed afterwards.

Optimised lysis solution (1% Triton X-100, 1% NP-40, 0.1 mM EDTA, 2% Chelex-100): The lysis solution used in our device has been optimised several times, which ensured a certain degree of lysis efficiency of the samples, while not inhibiting the subsequent reactions. When used to treat complex samples (fecal and urine samples), the lysis solution could adsorb impurities from the samples and inhibit the activity of some enzymes to protect the target nucleic acids.

**Figure 2:**
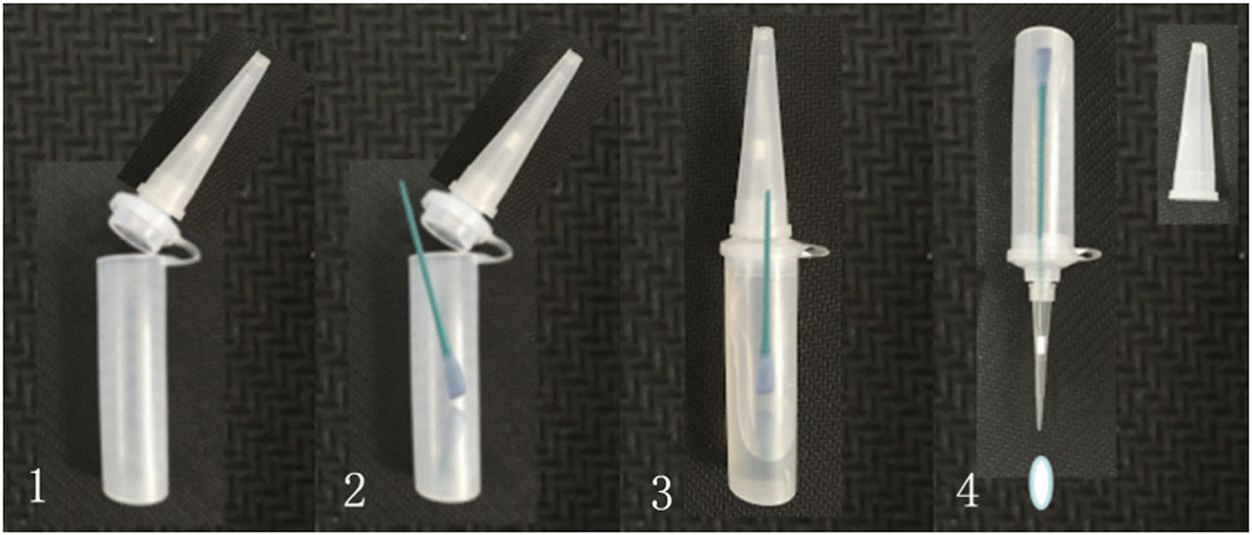
The operational procedure of a multi-functional sample treatment tube. 1. Open the hinged cover. 2. Place a cotton swab. 3. Heat the tube for lysis. 4. Remove the tip cover to squeeze out samples.

Simple operational process: As shown in Fig. 2, only four simple steps are required to complete the process from a sampling step to a nucleic acid extraction step—open the hinged cover, place a cotton swab containing the sample inside the tube, heat the end ‘a’ for activating the lysis solution, and remove the sample introduction-tip cover in the end ‘b’ to squeeze out sample droplets. The heat lysis was conducted at 95 °C for 10 min. The process was completed within 15 min, providing nucleic acids from the sample to be used for amplification in the downstream process.

The multi-functional sample treatment device mentioned above was used to extract nucleic acids from a sputum sample. The extracted nucleic acids were subjected to quantitative fluorescence PCR (CapitalBio, China), and the results were then compared to those obtained for the same sputum sample by two commercial kits—TaKaRa Mini BEST Bacteria Genomic DNA Extraction Kit Ver.3.0 and QIAamp DNA Mini Kit, as shown in Table 1. The data in the table clearly show that our device had nucleic acid extraction efficiency similar to that of the commercially available kits; hence, the device may be used in field settings for rapid nucleic acid extraction from *M*. *tuberculosis*.

**Table 1:** Comparison of qPCR results of extracted nucleic acids from *Mycobacterium tuberculosis*.

Sample	Sample treatment method	1(C_t_value)	2(C_t_value)	3(C_t_ value)	4(C_t_ value)
Pure bacterial liquid inoculum	Takarakit	23.76	27.36	30.62	34.45
		24.14	27.54	30.89	33.57
	Multi-functional sample treatment tube	24.97	28.57	31.2	33.84
		25.04	28.67	31.18	34.45
Simulated sputum sample	Qiagen kit	25.33	27.7	30.69	N/A
		25.21	27.14	30.17	N/A
	Multi-functional	25.09	27.33	30.7	N/A
	sample treatment tube	24.84	27.24	31.46	N/A

Note: 1, 2, 3, and 4 denote the count of different colonies of *M*. *tuberculosis*. 1: 10^7^ CFU/ml; 2: 10^6^ CFU/ml; 3: 10^5^ CFU/ml, 4: 10^4^ CFU/ml. Each sample had a parallel control.

## Preparation of the ***M. tuberculosis*** detection tube

### Nucleic acid amplification

We invented PSR^10,11^, a new *in vitro* isothermal nucleic acid amplification method, and own all the intellectual property rights. In this method, only two primers were required, each with the same forward sequence at the 5′ end, or one with a forward sequence and the other with a reverse sequence at the 5′ end, enabling the primers to use themselves as templates to substantially replicate the target nucleic acid segment. Using *Bst* DNA polymerase (large fragment), the target nucleic acid sequence could be replicated in a large quantity at a constant temperature in the range of 60–65 °C within 30–40 min, which is highly suitable for a rapid test in bed or field settings. The whole PSR reaction requires only one enzyme, greatly simplifying the procedure and reducing the expenses.

### Mechanism underlying the colour development reaction

The detection result was observed using a colour development reaction associated with pH change as elaborated below. When DNA polymerase adds a free deoxyribo nucleotide molecule to a nascent DNA strand, a hydrogen ion is produced as the byproduct, which means that the pH of the reaction system decreases continuously as more hydrogen ions are produced with the progression ofthe PSR reaction. The reaction mixture solution, in absence of the three heat-unstable components—enzyme, primers and dNTP—was heat-stable, and was termed in this study as an ionic solution, and could be prepared by mixing several components—20 mM (NH_4_)_2_SO_4_, 100 mM KCl, 16 mM MgSO_4_, 0.2% Tween 20, and 1.6 M betaine—and adjusting the pH to approximately 8.0 with 1% KOH. To simplify the experimental procedure, we used a solid sealant in advance to seal a 30-µL aliquot of the ionic liquid in the reaction tube. In the detection process, two drops (approximately 20 µL containing the sample) of the lysis solution (pH~11) were added to the tube cover. The vitrified mixture of the enzyme, primers, and dNTP was acidic. The mixture of ionic liquid, lysis solution, and vitrified mixture generated a system with a pH of approximately 8.8. After placing it in the tube, the ionic liquid was isolated from air by a sealant on the top of the liquid. On the tube cover there was a vitrified mixture of four components—enzyme, primers, dNTP, and cresol red-phenol red, with the mixture showing a dark yellow colour. After the colour development reaction was completed, the appearance of a yellow colour would indicate a positive result while the appearance of a red colour would indicate a negative result.

### Vitrification of heat-unstable components

The principles of vitrification^12^ are briefly introduced below. When some substances experience a temperature equal to or lower than their glass transition temperature (Tg), they form a glass in which there are almost no molecular motions or diffusions, and therefore, the substance can be preserved for a long time at ambient temperatures. During the vitrification process, the substances do not crystallise, but form an extremely sticky ‘super cooled’ liquid, which still shows molecular randomness—a characteristic of liquids—and therefore, glass is sometimes referred to as an’amorphous solid’ to be distinguished from a real solid. When heat-unstable components are in a vitrified status, their molecules are in an ‘inactive’ status, and therefore, the molecular activity can be preserved at ambient temperatures. Vitrification has found many practical applications, such as seed preservation. Air-cured mature seeds planted after 2–5 years of preservation under seal may still be able to germinate. The seed preservation process is a natural vitrification process.

The following components were added to the tube cover: 10U *Bst* DNA polymerase without glycerol, two 50-µM primers (each 0.8 µL) for Ft and Bt, respectively, two 50-µM primers (each 0.4 µL) for IF and IB, respectively, vitrification liquid (1.0 µL), cresol red-phenol red indicator (1.0 µL), and 3.5 µLdNTP (each, 10 mM), and the resulting system was then placed over a stove for 2–3 h to ensure that the vitrification was complete. The target we used for *M*. *tuberculosis* detection is the insertion sequence IS6110 (Genbank No.: CP002992.1). The PSR primers for amplification of target sequences are shown in Table 2.

**Table 2:** Polymerase spiral reaction primers for the detection of *M*. *tuberculosis*.

Primer	Sequence(5′ to 3′)
Ft	GTGCCCGCAAAGTGTGGCTAACTTGCGCGATGGCGAACTCA
Bt	GTGCCCGCAAAGTGTGGCTAACTTAGTTTGGTCATCAGCCGTTC
IF	ACGCGGCTGATGTGCT
IB	AGGTGGCCAGATGCACC

### All-in-one test kit for the detection of *M*. *tuberculosis*

**Figure 3:**
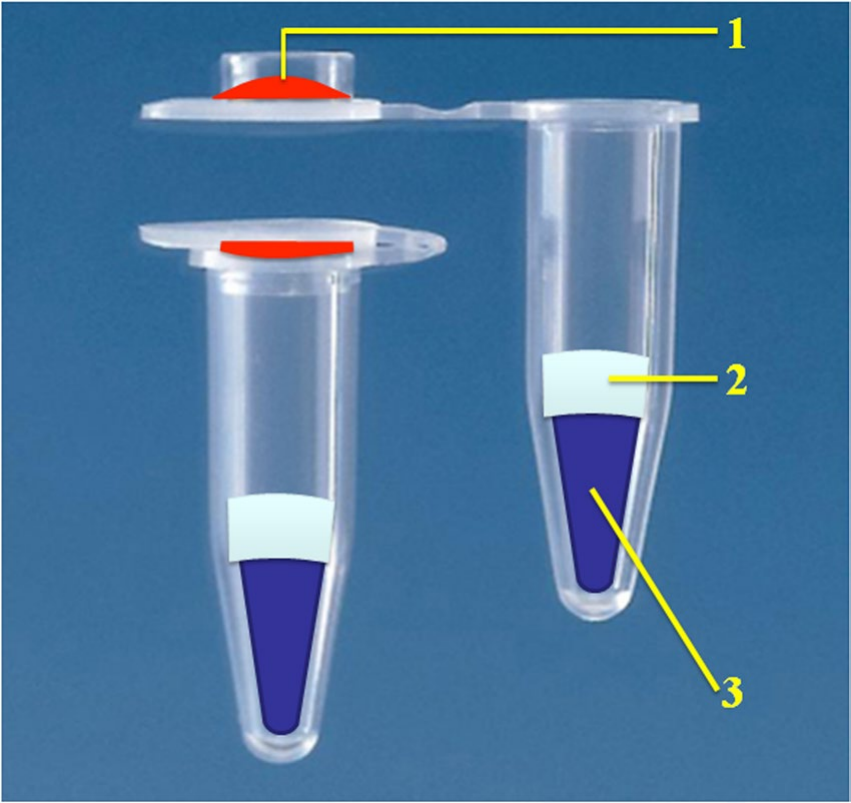
Schematic illustration of the all-in-one package. 1. Enzymes and other heat-unstable components. 2. Isolating substances to prevent contamination. 3. A heat-stable reaction system.

We employed the vitrification method to vitrify the heat-unstable components (enzymes, dNTP, and primers for *M*. *tuberculosis*) onto a reaction tube cover, placing the heat-stable components (the ionic liquid) at the bottom of the reaction tube, and sealing them with paraffin wax (melting point 50 °C), thereby generating an all-in-one test tube that was pre-filled with an entire reaction system, as shown in Fig. 3. Such package had three advantages: 1) nucleic acid test agents could be preserved at a room temperature for more than 12 months; 2) the test operation was simplified, with only the need to add sample nucleic acids to the tube cover; 3) sealing by paraffin wax prevented contamination ofthe amplification products.

**Figure 4:**
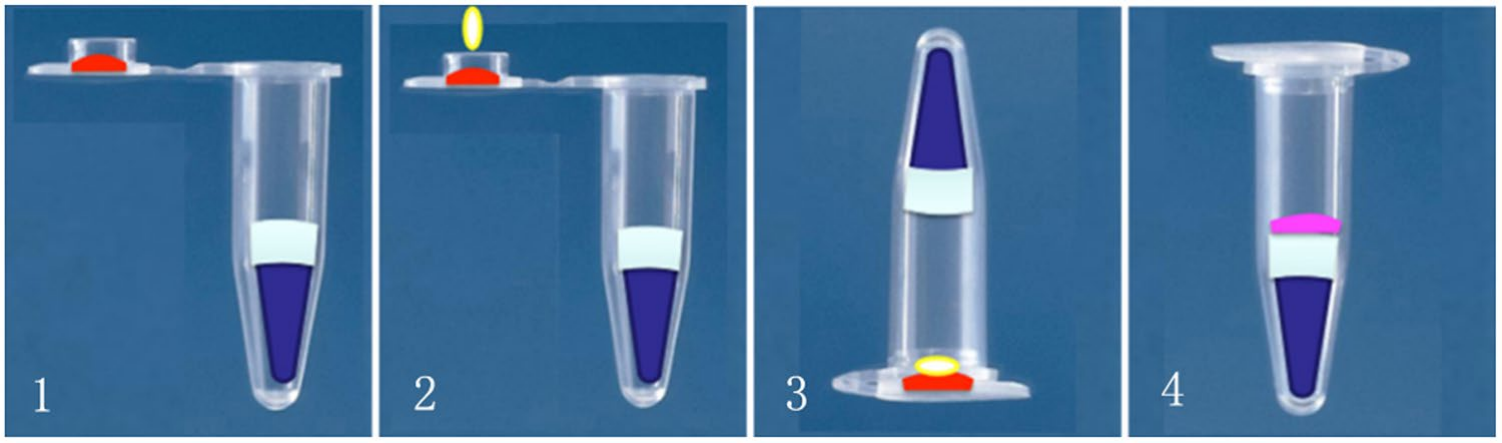
Operational steps of the all-in-one kit package. 1. Open the tube cover. 2. Add the sample to the cover. 3. Close the cover and reverse the tube. 4. Swing the sample liquid to the bottom.

The operational procedure of the all-in-one kit package could be completed in four simple steps as follows: open the tube cover, add the sample to the cover, close the cover, reverse the tube, and swing the sample liquid to the tube bottom, as shown in Fig. 4.

## Experimental Results

### Thermal stability test of the all-in-one kit for *M*. *tuberculosis*

**Figure 5:**
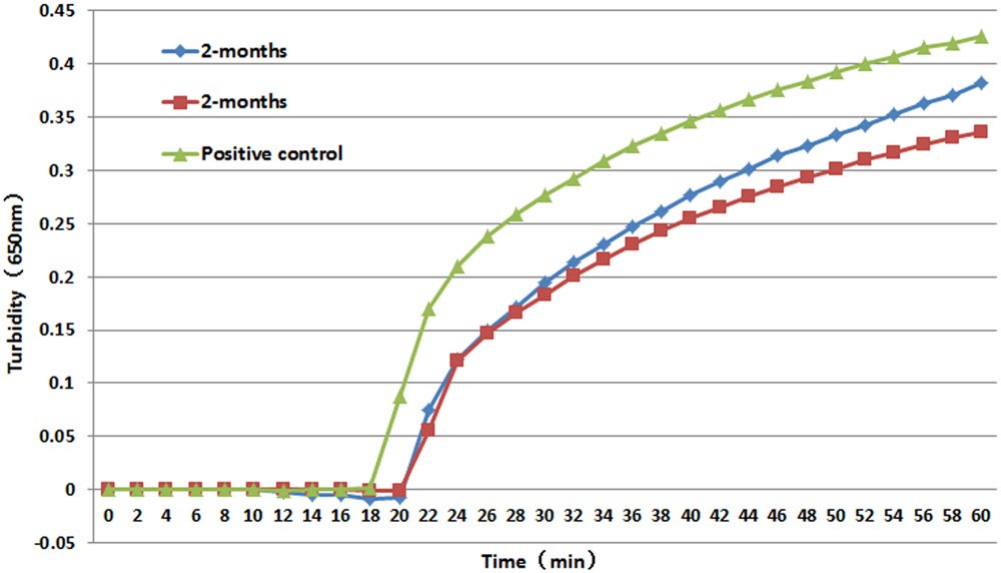
Thermal stability test for the all-in-one kit package at 50 °C. Two ‘2 months’ lines indicate two independent repeat tests.

We vitrified the PSR primers of *M*. *tuberculosis*, *Bst* DNA polymerase, and dNTP (heat-unstable components) onto the reaction tube cover and sealed the ionic liquid at the tube bottom by using paraffin wax; based on such a packaging technology, we successfully developed an all-in-one kit to be used for rapid detection of *M*. *tuberculosis*. The kit was preserved at 50 °C for two months, and then compared with an *M*. *tuberculosis* test kit that was not made into an all-in-one type of package, and the comparison of results is shown in Fig. 5. As shown in the figure, the enzyme and other heat-unstable components did not show a change in their activity after the 2-month storage at 50 °C, a storage equivalent to room temperature of 1–2 years in terms of the effect of storage on the activity.

### Specificity test of the all-in-one kit for *M*. *tuberculosis*

**Figure 6:**
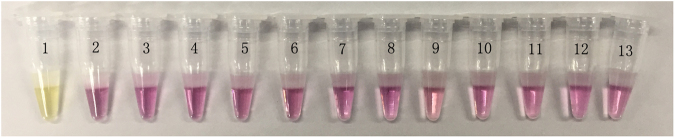
Specificity test results of the all-in-one kit for Mycobacterium tuberculosis. 1: M. tuberculosis; 2: Listeria monocytogenes; 3: Staphylococcus aureus; 4: Shigella flexneri; 5: Shigella sonnei; 6: Shigella dysenteriae CMCC(B)51105; 7: Salmonella typhimurium ATCC14028; 8: Salmonella enteritidis CMCC(B)50335; 9: Vibrio parahemolyticus ATCC17802; 10: Escherichia coli O157; 11: Escherichia coli NCTC12900; 12: Pseudomonas aeruginosa CMCC(B)10104; 13: negative control.

**Figure 7:**
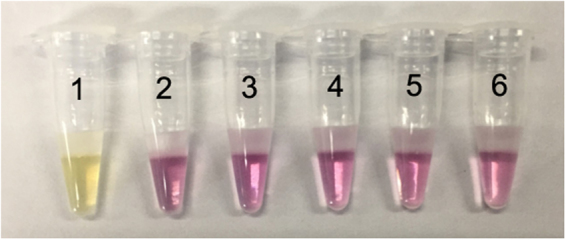
Specificity test results of the all-in-one kit for Mycobacterium tuberculosis. 1: M. tuberculosis; 2: Mycobacterium kansasii; 3: Mycobacterium scrofulaceum; 4: Mycobacterium avium-intracellulare; 5: Mycobacterium ulcerans; 6: negative control.

To test the specificity of the all-in-one kit for *M*. *tuberculosis*, we employed 11 bacteria and 4 non tuberculous mycobacteria (NTM) species—*Listeria monocytogenes*, *Staphylococcus aureus*, *Shigella flexneri*, *Shigella sonnei*, *Shigella dysenteriae* CMCC(B)51105, *Salmonella typhimurium* ATCC14028, *Salmonella enteritidis* CMCC(B)50335, *Vibrio parahemolyticus* ATCC17802, *Escherichia coli* O157, *Escherichia coli* NCTC12900, *Pseudomonas aeruginosa* CMCC(B)10104, *Mycobacterium kansasii*, *Mycobacterium scrofulaceum*, *Mycobacterium avium-intracellulare*, *Mycobacterium ulcerans*—as controls, and the experimental results are shown in Figs 6 and 7. As shown in the Fig. 6, positive results were obtained only when *M*. *tuberculosis* was used as the template, demonstrating that the all-in-one kit had a good specificity.

### Detection limit of the all-in-one kit for *M*. *tuberculosis*

**Figure 8:**
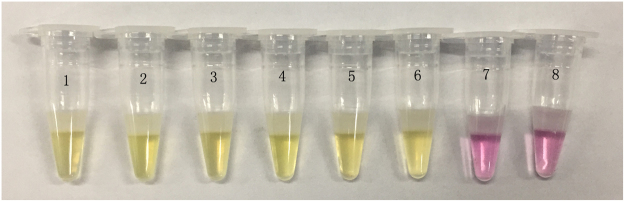
Detection limit test results of the all-in-one kit for *M*. *tuberculosis*. Nucleic acid concentrations in the tubes 1–7 were 92 ng/µL, 9.2 ng/µL, 920 pg/µL, 92 pg/µL, 9.2 pg/µL, 0.92 pg/µL, and 0.092 pg/µL, respectively, while the tube 8 was maintained as a negative control.

To test the detection limit of the all-in-one kit for *M*. *tuberculosis*, we used the *M*. *tuberculosis* genome as the testing object, and made a series of 10-fold dilutions of the total DNA solution, generating nucleic acid concentrations of 92 ng/µL, 9.2 ng/µL, 920 pg/µL, 92 pg/µL, 9.2 pg/µL, 0.92 pg/µL, and 0.092 pg/µL, respectively. The test was conducted using double distilled water as the negative control. The results, as shown in Fig. 8, indicated that the limit of detection (LOD) of this all-in-one kit for *M*. *tuberculosis* was 0.92 pg/µL, identical to the LOD determined with the fluorescence quantification, showing that the two methods had a similar detection limit.

### Application of the all-in-one kit for *M*. *tuberculosis* using sptum samples

The efficiencies of the prepared kit for *M*. *tuberculosis* and a fluorescence quantification kit (CapitalBio, China) were compared. A total of 131 clinical samples were collected from the patients from Institute of Tuberculosis, 309 Hospital of PLA, and a comparison between the two methods was conducted. The results, as shown in Table 3, indicated that the concordance rate between the two kits was 91.6%.

**Table 3:** Test results of *M*. *tuberculosis* in hospital 309. The qPCR was carried out by the CapitalBio fluorescence quantification kit. The PSR was carried out by the GenePop method.

	PSR-positive	PSR-negative	total
qPCR-positive	58	3	61
qPCR-negative	8	62	70
total	66	65	131

### Ethical approval

The study was carried out in accordance with the recommendations of the ethics committee of the Academy of Military Medical Sciences. The protocol was approved by the ethics committee of the Academy of Military Medical Sciences.

### Informed consent

Informed consent was obtained from all individual participants included in the study.

## Discussion

Currently, all the nucleic acid kits have to be stored and transported at low temperatures, thus increasing the cost and risk, and a problem at any stage in the transport chain would make the kit reliability questionable. We extracted proper vitrification liquids from seeds (a preservation process resembling the process of weeds being air cured), used the liquids to preserve the *Bst* DNA polymerase and the Taq DNA polymerase, and finally achieved storage of these two polymerases at ambient temperatures. Our current experimental results showed that the activity of these two polymerases preserved at ambient temperatures would remain unchanged for 1–2 years. We successfully employed this technology to the PSR, LAMP, and qPCR tests. Except for 0–3 min delay in the reaction time, there was no change in the specificity and detection limit of these tests.

Our ultimate objective was to enable nucleic acid diagnosis at any time and at any location. To realise such an objective, we—after overcoming a series of problems related to isothermal amplification, storage at ambient temperatures, all-in-one kit development, sample treatment, and prevention of contamination—successfully developed a novel, portable, and rapid nucleic acid test platform (GenePop) that was characterized by storage at room temperature, immediate test upon decapsulation, operation with ease, and straightforward test results, providing an alternative for the rapid diagnosis of pathogenic microbes. The technology platform was composed of four modules—cotton swabs for sampling, sample treatment tube, detection tube, and thermostatic apparatus. With five simple operation steps—using cotton swabs for sampling, heating the tube for DNA denaturation, squeezing out two droplets, amplifying the DNA at a constant temperature, and observing the results—the personnel may complete the process within approximately 1 h. The test platform did not require the use of any other device or preparation of any solution, and could work even after a power outage and water supply failure. The platform had the following characteristics:

1. It had good portability and was composed of only four parts.The platform only weighed 4.5 kg, which ensured that it would be carried and transported with great ease.
2. The operation was simple. The whole operation process consisted of five simple steps as depicted in Fig. 9, requiring no other device to be used or any solution to be made. An untrained person may learn the complete process within 30 min, which makes this platform very suitable for the personnel working at grass-root units.

**Figure 9:**
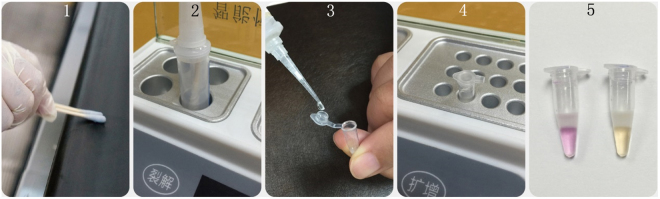
Operational steps of the novel kit for rapid nucleic acid test in field settings. 1. Use cotton swabs for sampling. 2. Heat the tube for DNA denaturation. 3. Squeeze out two droplets. 4. Amplify the DNA at a constant temperature. 5. Observe the results.
3. It could be stored at ambient temperatures. By employing the most cutting-edge, ambient temperature vitrification technology to immobilise all the heat-unstable components of the reaction solution onto the reaction tube cover, we could preserve those components for 12 months at room temperatures and transport them at temperatures of no more than 35 °C, thereby overcoming the limitations due to cold-chain transport and low temperature storage.
4. The amplification was conducted at a constant temperature. We employed the polymerase spiral reaction (PSR) method, for which we own all the intellectual property rights.
5. The process could be conducted in a timely manner. The sample treatment and the amplification steps took 15 min and 50 min, respectively, and therefore the complete process would take approximately 1 h, which was 1–3 h shorter than the time required for quantitative fluorescence PCR.
6. The platform had a low cost. With the ambient temperature vitrification technique, the platform had a low consumption of electric power and thereby a low production cost, making it very suitable for wide use in developing countries.
